# The Salaries of Hospital Secretaries

**Published:** 1896-01-18

**Authors:** 


### EDITOR'S LETTER-BOX.

[Our correspondents are reminded that proxility is a great bar to publication, and that brevity of style and conciseness of statement greatly facilitate early insertion.]

### THE SALARIES OF HOSPITAL SECRETARIES.

"A Hospital Secretary of Twenty-five Years" writes: In your account of the meeting at the Great Northern Central Hospital, held on December 30th, the chairman, Mr. Murdoch, is reported to have said "That Mr. Grant's salary only averaged £75 0 per annum, including everything. Not so much as was paid to their secretaries by other London hospitals." As this statement is absolutely incorrect, and must be prejudicial to the hospitals in general, I think some notice should be taken of it. From information at my disposal I can, without hesitation, say that there are not half a dozen hospital secretaries in London that receive anything like such remuneration.

